# Prediction of Safety Risk Levels of Benzopyrene Residues in Edible Oils in China Based on the Variable-Weight Combined LSTM-XGBoost Prediction Model

**DOI:** 10.3390/foods12112241

**Published:** 2023-06-01

**Authors:** Cheng Hao, Qingchuan Zhang, Shimin Wang, Tongqiang Jiang, Wei Dong

**Affiliations:** 1 National Engineering Research Centre for Agri-Product Quality Traceability, Beijing Technology and Business University, Beijing 100048, China; hc2680400194@163.com (C.H.);; 2 China Food Flavor and Nutrition Health Innovation Center, School of E-Business and Logistics, Beijing Technology and Business University, Beijing 100048, China

**Keywords:** risk assessment, LSTM, XGBoost, risk prediction, edible oil, BaP

## Abstract

To assess and predict the food safety risk of benzopyrene (BaP) in edible oils in China, this study collected national sampling data of edible oils from 20 Chinese provinces and their prefectures in 2019, and constructed a risk assessment model of BaP in edible oils with consumption data. Initially, the k-means algorithm was used for risk classification; then the data were pre-processed and trained to predict the data using the Long Short-Term Memory (LSTM) and the eXtreme Gradient Boosting (XGBoost) models, respectively, and finally, the two models were combined using the inverse error method. To test the effectiveness of the prediction model, this study experimentally validated the model according to five evaluation metrics: root mean square error (RMSE), mean absolute error (MAE), precision, recall, and F1 score. The variable-weight combined LSTM-XGBoost prediction model proposed in this paper achieved a precision of 94.62%, and the F1 score value reached 95.16%, which is significantly better than other neural network models; the results demonstrate that the prediction model has certain stability and feasibility. Overall, the combined model used in this study not only improves the accuracy but also enhances the practicality, real-time capabilities, and expandability of the model.

## 1. Introduction

Edible oil plays an indispensable role in daily life, enhancing the taste of food when frying and providing us with essential fatty acids. China is a large consumer of edible oil, with consumption reaching about 35.11 million tons in 2019 [1]. As China’s economy continues to develop steadily and rapidly, coupled with population growth, improved living standards, and accelerated urbanization, people’s consumption demand for edible oil will continue to grow steadily. In addition, the consumption landscape changed significantly with the onset of the COVID-19 epidemic. Quarantine and extended holiday initiatives were carried out across the country in response to the sudden spread of the epidemic. This led to many families stockpiling necessary living materials, including edible oil, which saw a corresponding increase in household consumption. Edible oil, being an essential item in Chinese kitchens, was particularly affected. In this article, edible oil generally refers to edible vegetable oil, edible animal oil, and edible oil products.

BaP is a polycyclic aromatic compound known for its toxic effects on the reproductive, blood, heart, nervous, and immune systems, and its ability to induce various cancers [2]. It is widespread in the environment, present in the atmosphere, surface water, sediment, soil, food, and fatty tissues, and can enter the food chain through various pathways, including biotransformation, impacting the metabolic processes of organisms. Some relevant studies have reported the addition of medicinal oil to extra virgin olive oil as a way to potentially mitigate BaP contamination, thereby improving health benefits and extending shelf life [3,4,5,6]. Therefore, the accurate determination of BaP content in edible oils is essential to assess their quality and safety and to safeguard the health of those who consume them [7].

The presence of BaP can be attributed to various sources [8,9]. One common route of BaP intake in humans is through the consumption of vegetable oil [10,11] which can contain a large amount of BaP-contaminated residues. Such contamination can occur during mechanical harvesting, transportation, and processing of the oilseeds, as these activities can lead to the oilseeds directly contacting pollutants, thus triggering the migration of BaP into the edible oil. In order to improve the oil yield and increase the aroma of the finished oil, such as peanut oil and sesame oil, the seeds are often fried before pressing, and the pressing process involves heating. This heating phenomenon during the vegetable oil pressing process, particularly when using the hot-pressing method, can produce a series of chemical reactions due to the high temperature, which may directly lead to the production of BaP, a carcinogen in edible oil [12]. In addition to direct contamination, there is also indirect contamination, such as asphalt contamination. Asphalt contains polycyclic aromatic hydrocarbons (PAHs), and farmers may contaminate soybeans by drying them on asphalt roads, resulting in oil pressed from these contaminated soybeans containing BaP [13,14].

Jiang et al. [15] conducted a health risk assessment of 75 randomly collected edible oils from Shandong Province, China, to evaluate the presence and hazards of PAH contamination, using Incremental Lifetime Cancer Risk (ILCR) as an evaluation metric. Their results indicated a widespread PAH contamination among the samples. Jang et al. [16] estimated the chronic daily exposure to BaP for the total population group and the consumer-only group using food consumption data from the fifth Korean National Health and Nutrition Examination Survey in 2011. Ref. [17] investigated 303 edible oils from Korea and used Margins of Exposure (MOEs) to understand the contamination levels of PAHs in them. Li et al. [18] used ILCR for assessing the risk of BaP in doughnuts. Gelavizh Barzegar et al. [19] used Monte Carlo simulations to characterize the daily intake MOEs and ILCR of edible oils sold in southwestern Iran. Bomi Kang et al. [20] employed MOEs to assess the risk of PAHs in Korean edible oils and found that despite the detection of PAHs, their effects on human exposure were not significant. The above studies only assessed the safety risk of BaP residues in edible oils by a single evaluation index and did not combine it with relevant food consumption data.

In recent years, the widespread use of deep learning prediction models in various fields, such as stock ticket price prediction [21,22,23], short-term traffic flow prediction [24,25,26], and urban air pollutant concentration prediction [27,28,29], has been enabled by the rapid development of artificial intelligence. Deep learning prediction models are also applicable to the requirements of food safety risk prediction. Jiang et al. [30] utilized deep learning to grade and predict the safety risk level of carbofuran pesticide residues in vegetables in China. Jiang et al. [31] proposed a risk prediction model for veterinary drug residues in freshwater products in China based on transform. Wang et al. [32] predicted the risk hazard of heavy metals in processed grain products using a voting integrated deep learning approach.

In this study, we used the national sampling data of BaP in edible oil in China in 2019 and the weekly consumption data of edible oil in each prefecture-level city as the basis for the in-depth calculation of evaluation indicators to build the data set. Firstly, we used the k-means algorithm to classify the evaluation indicators of edible oil by risk level, and then predicted the safety risk assessment indicators of edible oil in each prefecture-level city using the variable-weight combined LSTM-XGBoost prediction model, and classified these indicators according to the pre-defined risk level. The model proposed in this paper provides scientific and technical assistance for government regulatory authorities to monitor the safety of edible oils more effectively.

## 2. Materials and Methods

### 2.1. Materials

#### 2.1.1. Data Source

The data of BaP residues in edible oils in this study were obtained from the sampling data of the State Administration of Market Supervision of China 2019, covering 20 provinces, and contains a total of 12,826 samples. The consumption data of edible oils were obtained from the National Bureau of Statistics China Statistical Yearbook 2020. According to the national standard of China “Food Safety National Standard Limits of Contaminants in Food” (GB 2762-2017) [33], the maximum limit value of BaP in edible oil is 10 μg/kg.

#### 2.1.2. Data Pre-Processing

In this study, the substitution method recommended by the World Health Organization (WHO) [34] was used for samples below the detection limit of the method. When the proportion of non-detects was less than or equal to 60%, the results of all samples with detection results less than the LOD were calculated as 1/2 of the LOD. When the proportion of non-detects was greater than 60%, the results of all samples with assays less than the LOD were calculated as the LOD. Since the data of samples with undetected BaP residues in this study were much lower than 60%, the undetected BaP levels in this study were calculated as 1/2 of the LOD value. LOD is the minimum limit of detection for BaP in edible oils according to the database used in this study and was taken as 0.2 μg/kg [35].

## 3. Food Safety Risk Grading Assessment and Prediction Model

Considering that this study focused solely on the contamination status of BaP in edible oils and based on the basic principles of food safety risk assessment and sampling data of food products, three assessment indexes were selected for the risk assessment of edible oils: ILCR, MOE, and the Nemerow Integrated Pollution Index (NIPI).

### 3.1. Evaluation Indicators

#### 3.1.1. Carcinogenic Risk Factor Method

*ILCR* [18,36,37,38] is the increased likelihood of developing cancer over a lifetime due to exposure to potential carcinogens. It is commonly used to assess the carcinogenic risk of a pollutant to humans and is calculated as:

(1) $${\mathrm{ILCR}}_{\mathrm{BaP}}=\frac{C\times \mathrm{TEF}\times \mathrm{Ir}\times \mathrm{Ep}\times \mathrm{SF}\times \mathrm{CF}}{\mathrm{BW}\times \mathrm{TA}}$$

where *ILCR* is the Incremental Lifetime Cancer Risk that evaluates the carcinogenic risk of contaminants to humans; C is the concentration of chemical contaminants in edible oil, and the median *BaP* content in edible oil, mg/kg, is used in this study; TEF is the toxicity equivalence factor of *BaP*, TEF = 1; Ir is the daily intake of edible oil, kg/d; Ep is the exposure frequency, 365 d/a; Ed is the duration of exposure over the average human lifespan, 70 a (25,550 d); SF is the *BaP* carcinogenicity slope factor, 7.3 kg·d/mg; CF is the conversion factor, 10^−6^ mg/ng; BW is the body weight, 60 kg; and TA is the average exposure time to chemical pollutants, 70 × 365 d.

With reference to the interval of potential carcinogenic risk between 10^−6^ and 10^−4^ proposed by the US Environmental Protection Agency (US EPA) for *ILCR* [39], the carcinogenic risk is divided into three categories: *ILCR* < 1 × 10^−6^, the carcinogenic risk is negligible; 1 × 10^−6^ ≤ *ILCR* ≤ 1 × 10^−4^, the carcinogenic risk is acceptable; and *ILCR* > 1 × 10^−4^, the carcinogenic risk is not negligible.

#### 3.1.2. Margin of Exposure Method

The *MOE* method [17,40] is used to evaluate the risk of *BaP* intake in the population, using the toxicity endpoint of primary hepatocellular carcinoma [41]. The calculation formula is as follows:

(2) $$\mathrm{MOE}=\frac{{\mathrm{BMDL}}_{10}}{\mathrm{Exp}}$$

where ${\mathrm{BMDL}}_{10}$ is the toxicity reference point, referring to the lower limit of the 95% benchmark dose confidence interval for a 10% incidence of hepatocellular carcinoma in animal toxicology experiments; this value is 0.07 mg/(kg·*BW*) for *BaP*.

(3) $$\mathrm{Exp}=\frac{F_{i}\times C_{i}}{\mathrm{BW}\times 1000}$$

where Exp refers to the daily intake of *BaP* per kilogram of body mass due to the consumption of edible oils; $F_{i}$ refers to edible oil consumption, kg/d; $C_{i}$ refers to the average content of *BaP* in edible oil, µg/kg; and BW refers to the body mass, taken as 60 kg. According to the “Report on the Nutrition and Chronic Disease Status of Chinese Residents (2015)” [42], the average body mass of Chinese residents is 60 kg.

According to the recommendations of the European Food Safety Authority (EFSA) [43]: *MOE* > 10,000 is of very low health risk and does not require attention in public health, while *MOE* < 10,000 is of some health risk and requires attention.

#### 3.1.3. Nemerow Integrated Pollution Index

The *NIPI* [44] reflects the characteristics of food contamination. Based on the sampling data of each province, the integrated contamination index was applied to calculate the contamination level of each sample, and the expression is as follows:

(4) $$\mathrm{NIPI}=\sqrt{\frac{P_{\max\left(i,j\right)}^{2}+P_{\mathrm{ave}\left(i,j\right)}^{2}}{2}}$$

where *NIPI* is the integrated pollution index of food j in province i; $P_{\max(i,j)}$ is the maximum value of pollution index of food j in province i; and $P_{\mathrm{ave}\left(i,j\right)}$ is the average value of pollution index $P_{i,j}$ of food j in province i.

(5) $$P_{i,j}=\frac{X_{i,j}}{S_{j}}$$

where $P_{i,j}$ is the contamination index of food j in province i; $X_{i,j}$ is the detection value of *BaP* content in food j in province i (mg/kg); and $S_{j}$ is the national limit standard of *BaP* in food j (mg/kg), taken as 0.01 mg/kg here.

### 3.2. Food Safety Grading Based on k-Means

**Figure 1:**
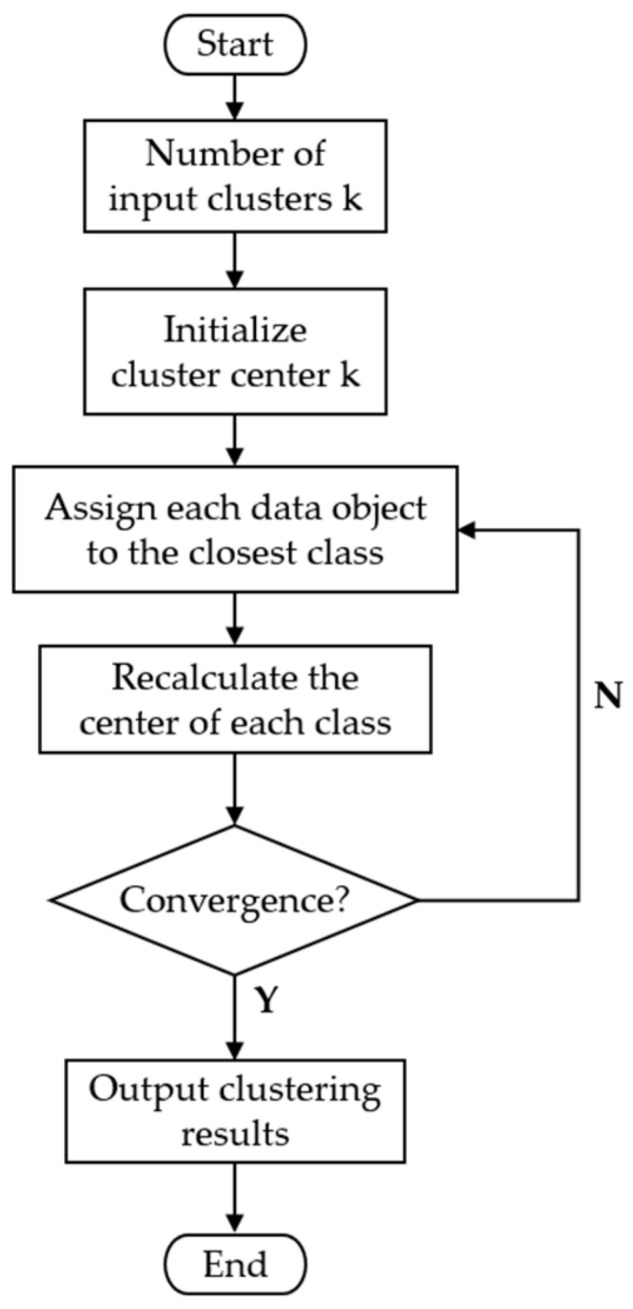
Flowchart of k-means clustering algorithm.

The k-means clustering algorithm is a commonly used method of cluster analysis that divides a data set into k clusters so that the data points within a cluster are as similar as possible, while those between clusters are as different as possible. In the food safety risk classification, the three evaluation indicators of edible oil (ILCR, MOE, and NIPI) were clustered and analyzed as a way to assess the safety risk level of edible oil in different prefecture-level cities in each province over time. By dividing the food samples into different clusters, food samples with similar characteristics can be placed in the same cluster, thus providing more refined and targeted control measures for food safety management. The specific process of the algorithm is shown in Figure 1.

(1) Select k objects from the data as the initial clustering centers;
(2) Calculate the distance from each clustering object to the cluster center to divide the clusters;
(3) Calculate each clustering center again;
(4) Calculate the standard measure function until the maximum number of iterations is reached and then stop; otherwise, continue the operation.

### 3.3. Food Safety Risk Level Prediction Model

**Figure 2:**
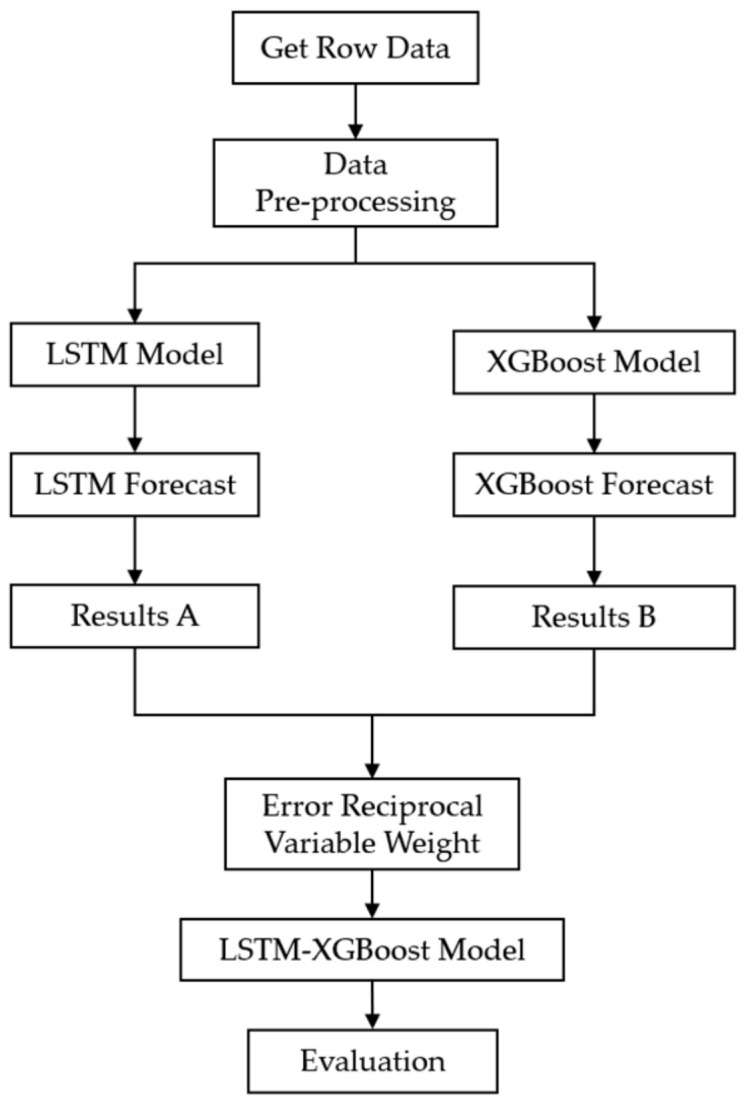
Flow chart of the variable-weight combined LSTM-XGBoost prediction model.

Considering that food sampling data are time-series and non-linear, we selected the LSTM model and XGBoost which have been frequently applied to such problems and have achieved better results. However, the LSTM model is a neural network model and the XGBoost model is a tree model. The principles of the two models differ greatly and the correlation of the prediction results is weak, so this study used these two theories to propose a food safety risk level prediction model that combines the LSTM model and the XGBoost model together to improve the overall prediction accuracy of the model, as shown in Figure 2. The results of this model are weighted using the inverse error method, a process that has been shown to significantly improve the accuracy of the combined model.

#### 3.3.1. LSTM Model

**Figure 3:**
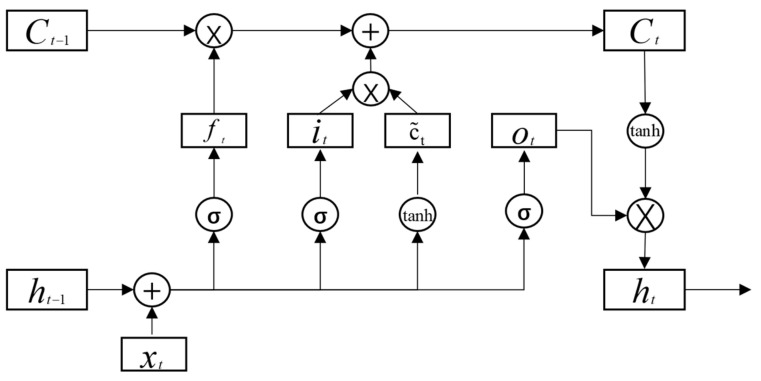
Hidden layer cell of LSTM model.

LSTM [45,46], also known as Long Short-Term Memory, is a variation of the traditional RNN, which can effectively capture the semantic association between long sequences and mitigate the gradient disappearance or explosion phenomenon compared with classical RNN. The structure of LSTM is complex; it includes an input layer, a hidden layer, and an output layer, each with many cells. Every cell in the hidden layer has memory cells, and the input gate, forgetting gate, and output gate collectively determine the output value. The internal structure of one of the hidden layer cells is shown in Figure 3 below.

In the figure, $x_{t}$ is the input value at moment t; $C_{t}$ is the memory cell at moment t; *h_t_* is the hidden cell at moment t; $f_{t}$ is the forgetting gate at moment t; $i_{t}$ is the input gate at moment t; $c_{t}$ is the candidate memory cell at moment t; $O_{t}$ is the output gate at moment t; and tanh and σ are both activation functions.

The forgetting gate, with inputs from the previous module output $h_{t-1}$ and the current time input data $x_{t}$, determines how much of $C_{t-1}$ is retained or forgotten from the previous cell module input. The input gate decides which new inputs can be retained to $C_{t}$, while the output gate, with control of $O_{t}$, determines what information is output and what needs to be transferred to the next module. The mathematical principle is to multiply the long-term memory input $C_{t-1}$ at t−1 by a forgetting factor $f_{t}$. The forgetting factor is calculated from the short-term memory $h_{t-1}$ as well as the event information $x_{t}$.

The formula for the calculation process of the forgetting gate is as follows:

(6) $$f_{t}=\sigma \left(W_{f}\cdot \left[h_{t-1},x_{t}\right]+b_{f}\right)$$

The input gate determines the amount of input $x_{t}$ saved to the unit state $C_{t}$ at the current moment, and it determines the corresponding new attribute information in this unit module for the attribute information discarded in the forgetting gate, and adds it to supplement the discarded attribute information. The mathematical principle is to accept the long-term memory $i_{t}$ from the forgetting gate and the short-term memory ${\tilde{C}}_{t}$ from the learning gate and then directly merge the two. The computational procedure for the input gate is given by:

(7) $$i_{t}=\sigma \left(W_{f}\cdot \left[h_{t-1},x_{t}\right]+b_{f}\right)$$

(8) $$\tilde{C_{t}}=\tanh\left(W_{C}\cdot \left[h_{t-1},x_{t}\right]+b_{C}\right)$$

(9) $$C_{t}=f_{t}*C_{t-1}+i_{t}*\tilde{C_{t}}$$

The current output gate $O_{t}$ determines the extent to which the state of the control unit $C_{t}$ is input to the current output value $h_{t}$. The mathematical principle is that $O_{t}$ is obtained using a Sigmoid function, and $O_{t}$ is multiplied by $\tanh\left(C_{t}\right)$ to obtain the final output $h_{t}$. The output is calculated by the formulas

(10) $$O_{t}=\sigma \left(W_{o}\cdot \left[h_{t-1},x_{t}\right]+b_{o}\right)$$

(11) $$h_{t}=o_{t}\times \tanh\left(C_{t}\right)$$

#### 3.3.2. XGBoost Model

The XGBoost model [25,47,48], which improves upon the Gradient Boosting Decision Tree (GBDT) model, utilizes a second-order Taylor expansion, unlike the traditional GBDT model which only uses a first-order Taylor expansion. This tends to complicate the GBDT model and makes it prone to overfitting. The XGBoost model incorporates features such as regularization, learning rate, column sampling, and the approximation of optimal splitting points, all of which help in preventing overfitting. XGBoost, being an integrated model comprising multiple trees, derives its prediction for a sample from the aggregate of the predicted values of each tree for that sample. The equation of the XGBoost model is as follows:

(12) $${\hat{y}}_{i}=\sum_{k=1}^{k}f_{k}\left(x_{i}\right)$$

where *k* is the total number of trees; $f_{k}\left(x_{i}\right)$ is the prediction result of the *k*th tree for the *i*th $x_{i}$; and ${\hat{y}}_{i}$ is the prediction result of the XGBoost model for the *i*th sample.

The objective function is expressed as.

(13) $$\mathrm{Obj}\left(\theta \right)=\sum_{i=1}^{n}l\left(y_{i},{\hat{y}}_{i}\right)+\sum_{k=1}^{k}\Omega \left(f_{k}\right)$$

where $l\left(y_{i},{\hat{y}}_{i}\right)$ denotes the training error of indicator sample $x_{i}$ in the original sample and $\sum_{k=1}^{k}\Omega \left(f_{k}\right)$ denotes the regularization term of the *k*th tree to prevent overfitting of the model.

(14) $$\Omega \left(f_{k}\right)=\gamma T+\frac{1}{2}\lambda \sum_{j=1}^{T}\omega_{j}^{2}$$

where T is the number of leaf nodes in each tree; $\omega_{j}^{2}$ is the weight of the *j*th leaf node; and γ and λ are coefficients, which need to be adjusted for the parameters in practical applications.

#### 3.3.3. LSTM Model Construction

In this study, LSTM and Dropout were chosen for the hidden layer to build two layers to prevent overfitting.

**Figure 4:**
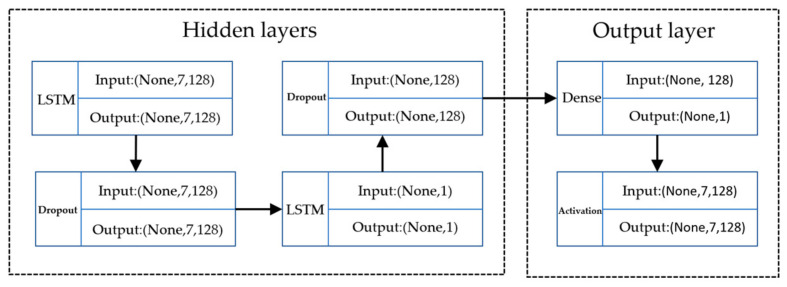
LSTM model construction.

As shown in Figure 4, the model, in terms of parameter settings, includes one input layer, two hidden layers, and one output layer. The default sigmoid activation function serves as the activation function, and the LSTM uses 7 as its Timesteps. The model selects Mean Absolute Error (MAE) as its loss function and adopts the Adam optimization algorithm for network training. The initial learning rate is set to 0.05, with a gradient threshold set to 1. The output layer employs a fully connected layer to reduce the dimensionality of the results. Upon obtaining the prediction data, the model performs inverse normalization, thereby obtaining the final prediction results.

#### 3.3.4. XGBoost Model Construction

The XGBoost model was built starting with the tuning of the tree parameters. The parameters are initialized based on the default values. The choice of parameters refers to the Mean Squared Error (MSE) as the loss function and Gamma as the objective function. When using the XGBoost model for temporal prediction, it is necessary to consider the following algorithmic parameters:

Learning_rate: The learning rate boosts the model’s robustness by reducing the weights at each gradient descent step, and the value typically ranges from 0.01 to 0.2. If the value is too low, it might cause underfitting in the model.

Gamma: A node only splits if the value of the loss function decreases post-split. Gamma determines the minimum decrease in the loss function required to split the node. The larger this parameter, the more conservative the algorithm will be, as a larger gamma value necessitates a more substantial decrease in the loss function before the node can split, reducing the likelihood of node splitting during tree generation.

Subsample: This parameter determines the proportion of random samples for each tree. By lowering this value, the algorithm will be more conservative and prevent overfitting. However, setting this value too low can lead to underfitting. It generally ranges between 0.5 and 1, with 0.5 representing an average sampling.

Colsample_bytree: This parameter is utilized to control the percentage of columns sampled randomly for each tree (each column corresponds to a feature).

Max_depth: This is the maximum depth of the tree, typically set between 3 and 10. A larger value allows the model to quickly identify the features of local samples, but it also increases the likelihood of overfitting and slows down the model’s training speed.

#### 3.3.5. Model Tuning Method

In the model tuning stage, this study employed ten-fold cross-validation combined with a grid search approach. Firstly, the cross-validation is used to assess the model’s performance, followed by a grid search to select the optimal parameters. The ten-fold cross-validation initially splits the edible oil data set into ten non-overlapping segments. Nine of these are used as training segments and one as a testing segment to enhance the model’s performance by reducing the variance in data partitioning.

Grid search, a commonly used method for parameter tuning, applies an exhaustive search method. After a set of hyperparameters is provided, an exhaustive search is carried out among all the hyperparameter combinations, aiming to select the optimal set from all combinations.

**Figure 5:**
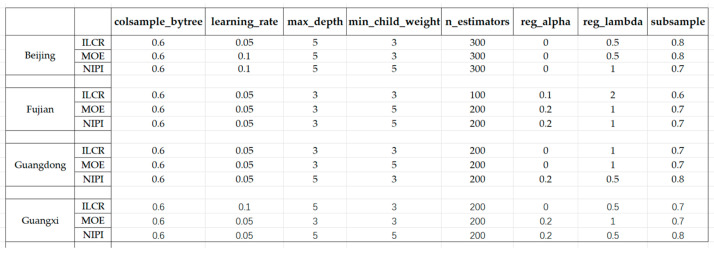
XGBoost model tuning results.

Given the differences in data across provinces, each province’s indicators were tuned separately during the tuning session. Following this, the experiment was conducted; the tuning results are depicted in Figure 5.

#### 3.3.6. The Inverse Error Method

The prediction results of each single model are obtained by the LSTM model and XGBoost Model, and for the analysis results, the following formula is applied for the inverse error method analysis to process the LSTM and XGBoost time series data.

(15) $$f_{t}=\omega_{1}f_{1t}+\omega_{2}f_{2t},t=1,2,\ldots ,n$$

(16) $$\omega_{1}=\frac{\varepsilon_{2}}{\varepsilon_{1}+\varepsilon_{2}}$$

(17) $$\omega_{2}=\frac{\varepsilon_{1}}{\varepsilon_{1}+\varepsilon_{2}}$$

where $\omega_{i}$ denotes the weight coefficient, $f_{\mathrm{it}}$ denotes the prediction data of LSTM and XGBoost, and $\varepsilon_{1}$ and $\varepsilon_{2}$ refer to the LSTM and XGBoost errors, respectively.

#### 3.3.7. The Variable-Weight Combined LSTM-XGBoost Prediction Model

**Figure 6:**
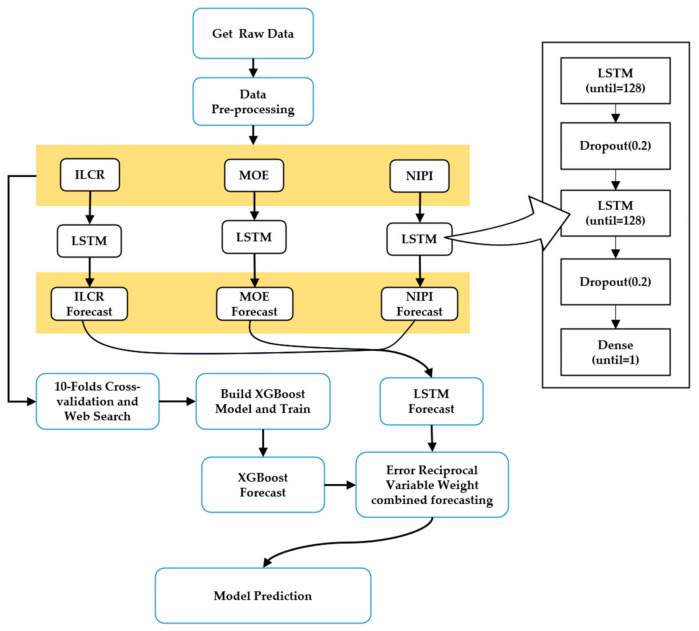
LSTM-XGBoost-based variable-weight combined prediction model.

Considering the substantial differences in principles between the LSTM model, which is a neural network model, and the XGBoost model, which is a tree model, and the relatively weak correlation between their prediction results, this study proposed integrating these two models using the inverse error method to improve the overall prediction accuracy. The primary process is as follows, and the corresponding flowchart is depicted in Figure 6.

(1) The pre-processed data are input into the LSTM and XGBoost models for predictive analysis, resulting in the prediction outcomes of each individual model.
(2) The prediction results of the obtained LSTM and XGBoost models are weighted and combined using the inverse error method to obtain the final prediction results of the combined LSTM-XGBoost model.
(3) The evaluation metrics RMSE and MAE are utilized to compare each individual model and the combined model.

## 4. Results and Discussion

### 4.1. Data Set Processing

The data set used in this study includes three evaluation indicators for edible oils (ILCR, MOE, and NIPI) for each prefecture-level city in 20 Chinese provinces in 2019. This data set contains a total of 12,826 records, and the total length of the time series for each city is 53 weeks. The data were divided in a 6:4 ratio for subsequent analysis or processing.

### 4.2. Experimental Environment

The computer configurations used for the experiments in this paper are shown in Table 1.

### 4.3. Model Evaluation Metrics

In order to scientifically measure the prediction effectiveness of this combined model, the five evaluation metrics were used to evaluate the model: *RMSE*, *MAE*, precision, recall, and *F*1 score [49,50].

*MAE* stands for Mean Absolute Error. It calculates the average absolute difference between the true and predicted values, preventing the errors from being cancelled out by positive or negative discrepancies. Generally, the lower the *MAE* value, the better the prediction ability of the model.

(18) $$\mathrm{MAE}=\frac{1}{n}\sum_{i=1}^{n}\left|y_{i}-{\hat{y}}_{i}\right|$$

*RMSE* is the abbreviation for Root Mean Square Error. This metric computes the square root of the averaged squared differences between predicted and actual observations. It is particularly sensitive to outliers and serves as a robust measure of the predictive capability of the model.

(19) $$\mathrm{RMSE}=\sqrt{\frac{1}{n}\sum_{i=1}^{n}{\left(y_{i}-{\hat{y}}_{i}\right)}^{2}}$$

where $y_{i}$ denotes the actual value of a single assessment indicator for week i; ${\hat{y}}_{i}$ denotes the predicted value of a single assessment indicator for week i; and n denotes the total number of data points to be measured.

Precision refers to the proportion of samples with a predicted value of 1 and a true value of 1 among all samples with a predicted value of 1.

(20) $$P=\frac{\mathrm{TP}}{\mathrm{TP}+\mathrm{FP}}$$

Recall, also known as the full rate, refers to the proportion of samples with a predicted value of 1 and a true value of 1 out of all samples with a true value of 1.

(21) $$R=\frac{\mathrm{TP}}{\mathrm{TP}+\mathrm{FN}}$$

where TP indicates the number of risk levels that the model correctly predicted and FP indicates the number of levels that the model predicted that are incorrectly predicted as being at that risk level. FN indicates the number of levels of that risk level that the model incorrectly predicted as other risk levels.

The *F*1 score, also known as the Balanced Score, is defined as the summed average of the precision and recall. To better evaluate the performance of the prediction model, this study used the *F*1 score as an evaluation criterion to measure the comprehensive performance of the model.

(22) $$F1=\frac{2\times P\times R}{P+R}$$

### 4.4. Edible Oil Safety Risk Classification and Assessment

#### 4.4.1. Risk Classification

In this study, the k-means algorithm was used to cluster and grade the three-evaluation metrics (*ILCR*, *MOE* and *NIPI*) in the assessment model. The elbow method was used to determine the value of k among them, and the core index of the elbow method is Sum of the Squared Errors (*SSE*).

(23) $$\mathrm{SSE}=\sum_{i=1}^{k}\sum_{p\in C_{i}}{\left|p-m_{i}\right|}^{2}$$

where $C_{i}$ is the *i*th cluster, p is the number of sample points in $C_{i}$, $m_{i}$ is the center of mass of $C_{i}$ (the mean of all samples in $C_{i}$), and *SSE* is the clustering error of all samples, which represents the performance of the clustering.

The core idea of the elbow method is that as the number of k clusters increases, the sample division becomes finer, and the degree of aggregation of each cluster gradually increases, causing the *SSE* to decrease progressively. When k is less than the true number of clusters, the decrease in *SSE* is significant as the increase in k substantially enhances the degree of aggregation of each cluster. However, when k reaches the true number of clusters, the returns on the degree of aggregation obtained by further increasing k rapidly diminish, leading to a sharp decrease in *SSE*, which then levels off as k continues to increase. Therefore, the graph of the relationship between *SSE* and k forms an ‘elbow’ shape, and the value of k corresponding to this elbow is considered to be the true number of clusters in the data.

**Figure 7:**
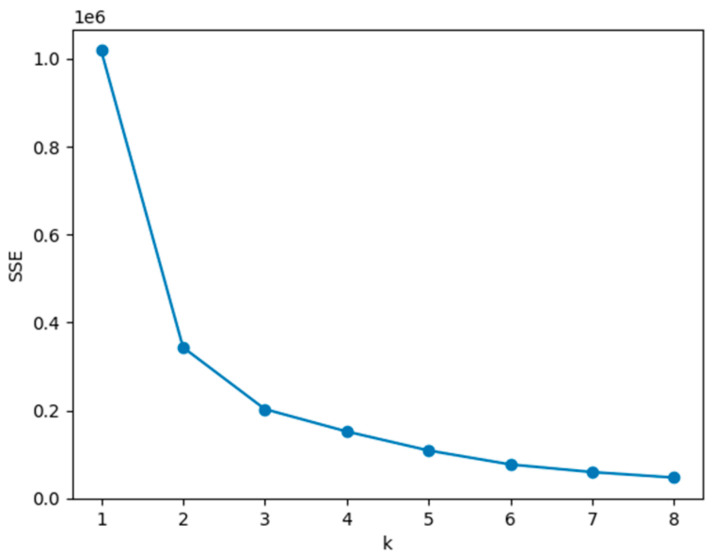
Elbow method to determine k value.

By observing Figure 7, we can see that the elbow corresponds to a k value of 3 (maximum curvature), and the *SSE* decreases quite significantly from 1 to 2 and from 2 to 3, while the *SSE* decreases very little from 3 to 4 and even after 4; therefore, the optimal k value should be 3. The results for each cluster center are shown in Table 2, and the distance of the cluster center from the origin is calculated based on the specific normalized index. Then, the risk level is defined as low, medium, or high based on the distance.

#### 4.4.2. Analysis of Grading Results

**Figure 8:**
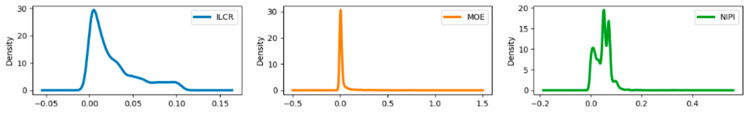
Probability density distribution of subgroup 1.

**Figure 9:**
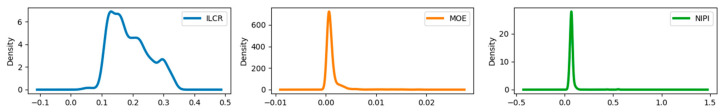
Probability density distribution of subgroup 2.

**Figure 10:**
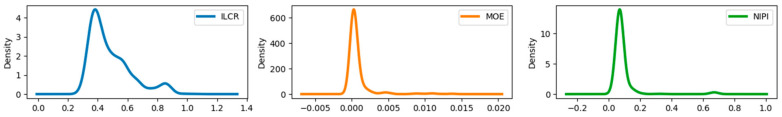
Probability density distribution of subgroup 3.

The distribution of indicators for the different security risk levels can be analyzed in Figure 8, Figure 9 and Figure 10.

Based on the above analysis, the following conclusions can be drawn.

(1) The ILCR values of subcluster 1 were distributed between 0 and 0.1, the MOE was less spaced and distributed between 0 and 0.001, and the NIPI values were concentrated between 0 and 0.1, indicating a low risk level;
(2) The ILCR values of subgroup 2 were distributed between 0 and 0.4, the MOE was distributed between 0 and 0.002, and the NIPI values were less spaced and concentrated between 0 and 0.2, indicating a medium risk level;
(3) Subgroup 3 had the highest ILCR values distributed between 0 and 0.9, the MOE values were distributed between 0 and 0.005, and the NIPI values had smaller intervals and were concentrated between 0 and 0.4, indicating the highest risk level.

The results of the above analysis show that, for the three indicators of the food safety risk assessment model, the k-means clustering algorithm can cluster the edible oil safety risks in each prefecture-level city at different time periods, and the samples could be divided into three groups, namely, subgroup 1 with a low risk level, subgroup 2 with an intermediate risk level, and subgroup 3 with the highest risk level. The *ILCR* values of subgroup 1 were small, the interval of MOE values was small, and the distribution of *NIPI* values was concentrated and small; the *ILCR* values of subgroup 2 were higher, the interval of MOE values was larger, and the *NIPI* values were at an intermediate level; the *ILCR* values of subgroup 3 were the largest, the interval of MOE values was also relatively the largest, and the *NIPI* values were at a high level. These results can provide targeted management measures and risk control programs for food safety regulatory authorities.

#### 4.4.3. Predicted Results of BaP Safety Risk Level in Edible Oil

In order to scientifically evaluate the prediction performance of the variable-weight combined LSTM-XGBoost prediction model in this paper, a comparative analysis was conducted. Considering that the predictive effect of this combined model will rely heavily on the predictive power of each single model, we conducted a comparative analysis of the LSTM model, the XGBoost model, and the variable-weight combined LSTM-XGBoost prediction model, and evaluated the prediction performance of the three models by comparing the errors between their prediction results and the actual values, and whether the variable-weight combined LSTM-XGBoost prediction model is superior to the individual models. We used a single-step prediction method with a step size of seven for the three-evaluation metrics of edible oils mentioned previously, and performed a preliminary analysis of the prediction results using RMSE and MAE.

**Figure 11:**
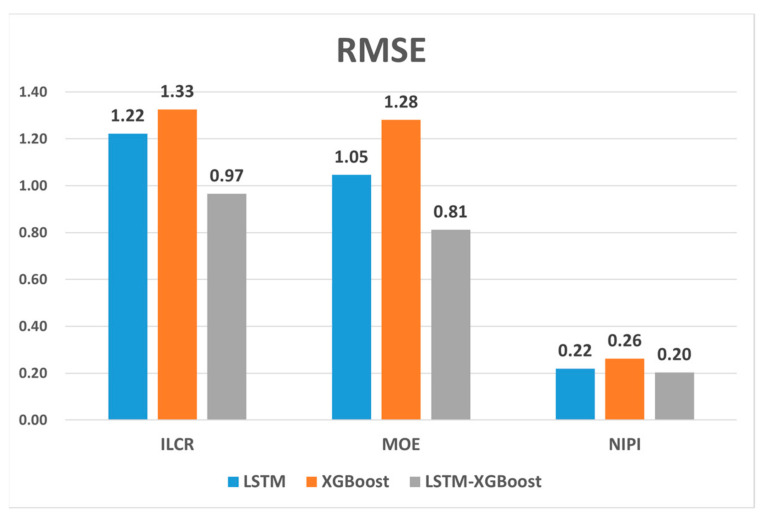
RMSE for ILCR, MOE, and NIPI indicators.

**Figure 12:**
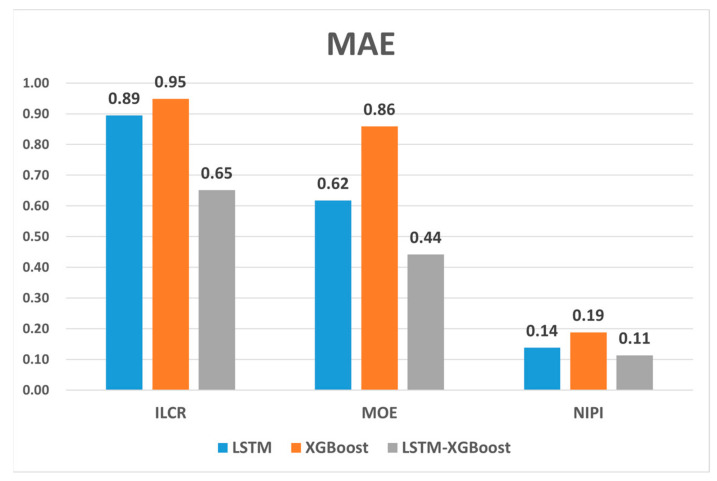
MAE for ILCR, MOE, and NIPI indicators.

Figure 11 and Figure 12 show the RMSE and MAE values of the food safety risk assessment indicators predicted by the three models. The result plots show that the evaluation indicators predicted by the combined model proposed in this paper had the smallest RMSE and MAE values. The RMSE measures the mean error between the model’s predicted and true values, while the MAE measures the mean absolute error between the model’s predicted and true values. Smaller RMSE and MAE values imply that the model has a higher prediction accuracy and is better able to adapt to changes in the test data set. We observed that the variable-weight combined LSTM-XGBoost prediction model had the smallest RMSE and MAE values compared to the LSTM and XGBoost models alone, indicating that the combined model can significantly improve the prediction accuracy.

After the models predicted the weekly ILCR, MOE, and NIPI indicators for different prefecture-level cities in each province, the distance between this rating indicator and the three clustering centers was measured, and the risk level rating for that week in that city was determined, and the precision (P%), recall (R%), and F1 scores (F1%) of the risk rating predicted by the three models were tallied, as shown in Table 3.

The experimental results show that the variable-weight combined LSTM-XGBoost prediction model proposed in this paper outperforms the other two models in terms of accuracy, and this model can provide a new approach to aid the government in regulating risky edible oils. In addition, the F1 value is significantly better than that of a single model. The F1 value shows that this model is able to balance the accuracy rate and recall rate, so the government can better capture potential food safety problems based on the model’s prediction results, target and strengthen the regulation of specific products, specific supply chain links or specific regions, and optimize resource allocation, thus improving the overall food safety level.

## 5. Conclusions

BaP is one of the most representative carcinogens among the more than 20 known carcinogenic PAHs. Fats and oils containing PAHs can intensify absorption in the intestinal tract, thus posing a great threat to human health. To reduce dietary intake of BaP, we should maintain a balanced and diverse diet that includes a variety of fruits and vegetables, avoid excessive intake of grilled meats, especially charcoal-grilled and smoked meats, and remove burnt parts of foods. Whenever possible, choose fats and oils rich in monounsaturated fatty acids (e.g., canola and olive oils) and polyunsaturated fatty acids (e.g., corn and soybean oils).

In order to thoroughly assess the safety risk of BaP in edible oils in China and to carry out precise regulation to effectively protect the health and safety of residents’ food, we introduced an innovative prediction model, namely, the variable-weight combined LSTM-XGBoost prediction model. This model combines two leading algorithms, LSTM and XGBoost, combining their respective strengths to improve the prediction accuracy; the LSTM algorithm is a modeling approach that is well suited for handling serial data, which can effectively capture long-term dependencies in time series; the XGBoost algorithm is effective at handling nonlinear relationships and high-dimensional data. Therefore, the variable-weight combined LSTM-XGBoost prediction model has the ability to better handle data with time-series and high-dimensional features, which helps to improve the accuracy of prediction. The advantages of this combined model are further enhanced by the adoption of the inverse error method, which adjusts the combined weights of the model by optimizing the inverse of the prediction error so that the prediction results of both algorithms can be optimally combined to further improve the prediction accuracy.

Experimentally, by comparing the variable-weight combined LSTM-XGBoost prediction model with the LSTM and XGBoost models alone, we found that the former performed better in terms of prediction accuracy, as evidenced by the lowest values of two crucial metrics, RMSE and MAE. Thus, the variable-weight combined LSTM-XGBoost prediction model is undoubtedly an efficient and effective way to combine algorithms to provide more accurate prediction results for data with time-series and high-dimensional characteristics. More importantly, this model also shows strong utility in food safety risk assessment, and the experimental results show that its F1 score was as high as 95.16%, which is a good balance of accuracy and recall. This means that the model is able to meet the high demand of food regulatory authorities to monitor the safety of edible oils in different prefecture-level cities in each province and strengthen early warnings and control of food safety, which also provides guidance in optimization of the allocation of resources, thereby more effectively preventing the occurrence of food safety risks.

## Figures and Tables

**Table 1:** Experimental platform and environmental parameters.

Computer Information	Operating System	Windows 10 64-bit
	CPU	AMD Ryzen 7 5800H with Radeon Graphics 3.20 GHz
	GPU	Nvidia GeForce GTX1650
	Memory	16 GB
Toolkit	Python 3.7.11	Numpy 1.18.5
		Pandas 1.2.2
		Keras 2.9.0
		Torch 1.8.3
		Matplotlib 3.5.3

**Table 2:** Clustering centers and ranking of the 3 clusters.

Category	ILCR	MOE	NIPI	Sample Size	Risk Level
1	0.026709	0.021994	0.047369	9177	Low
2	0.911746	0.001170	0.074586	3095	Medium
3	0.483147	0.000756	0.089037	554	High

**Table 3:** Experimental results of risk level prediction.

Model	P%	R%	F1%
LSTM	81.23%	78.66%	79.92%
XGBoost	80.08%	82.42%	82.19%
LSTM-XGBoost	94.62%	95.71%	95.16%

## Data Availability

The data presented in this study are available on request from the corresponding author. The data are not publicly available due to the data were available with the permission of the State Administration for Market Regulation Statistics.
